# Prognosis after hepatic resection of patients with hepatocellular carcinoma related to non-alcoholic fatty liver disease: meta-analysis

**DOI:** 10.1093/bjsopen/zrac167

**Published:** 2023-02-20

**Authors:** Jia-Yong Su, Zhu-Jian Deng, Yu-Xian Teng, Ye Xin Koh, Wan-Guang Zhang, Ming-Hua Zheng, Si Xie, Rong-Rui Huo, Chao-Jing Chen, Liang Ma, Jian-Hong Zhong

**Affiliations:** Hepatobiliary Surgery Department, Guangxi Medical University Cancer Hospital, Nanning, China; Hepatobiliary Surgery Department, Guangxi Medical University Cancer Hospital, Nanning, China; Hepatobiliary Surgery Department, Guangxi Medical University Cancer Hospital, Nanning, China; Department of Hepatopancreatobiliary and Transplant Surgery, Singapore General Hospital, Singapore; Hepatic Surgery Center, Tongji Hospital, Tongji Medical College, Huazhong University of Science and Technology, Wuhan, China; NAFLD Research Center, Department of Hepatology, the First Affiliated Hospital of Wenzhou Medical University, Wenzhou, China; Hepatobiliary Surgery Department, Guangxi Medical University Cancer Hospital, Nanning, China; Hepatobiliary Surgery Department, Guangxi Medical University Cancer Hospital, Nanning, China; Hepatobiliary Surgery Department, Guangxi Medical University Cancer Hospital, Nanning, China; Hepatobiliary Surgery Department, Guangxi Medical University Cancer Hospital, Nanning, China; Hepatobiliary Surgery Department, Guangxi Medical University Cancer Hospital, Nanning, China; Key Laboratory of Early Prevention and Treatment for Regional High Frequency Tumor (Guangxi Medical University), Ministry of Education; Guangxi Key Laboratory of Early Prevention and Treatment for Regional High Frequency Tumor, Nanning, China

## Abstract

**Background:**

Whether the safety and efficacy of hepatic resection differ between patients whose hepatocellular carcinoma (HCC) is related to non-alcoholic fatty liver disease (NAFLD) or has other aetiologies is unknown. A systematic review was performed to explore potential differences between such conditions.

**Methods:**

PubMed, EMBASE, Web of Science, and Cochrane Library were systematically searched for relevant studies that reported hazard ratios (HRs) for overall and recurrence-free survival between patients with NAFLD-related HCC or HCC of other aetiologies.

**Results:**

The meta-analysis involved 17 retrospective studies involving 2470 patients (21.5 per cent) with NAFLD-related HCC and 9007 (78.5 per cent) with HCC of other aetiologies. Patients with NAFLD-related HCC were older and had higher body mass index (BMI), but were less likely to have cirrhosis (50.4 per cent *versus* 64.0 per cent, *P* < 0.001). The two groups suffered similar rates of perioperative complications and mortality. Patients with NAFLD-related HCC had slightly higher overall survival (HR 0.87, 95 per cent c.i. 0.75 to 1.02) and recurrence-free survival (HR 0.93, 95 per cent c.i. 0.84 to 1.02) than those with HCC of other aetiologies. In the various subgroup analyses, the only significant finding was that Asian patients with NAFLD-related HCC had significantly better overall survival (HR 0.82, 95 per cent c.i. 0.71 to 0.95) and recurrence-free survival (HR 0.88, 95 per cent c.i. 0.79 to 0.98) than Asian patients with HCC of other aetiologies.

**Conclusion:**

The available evidence suggests that patients with NAFLD-related HCC have similar perioperative complications and mortality, but potentially longer overall and recurrence-free survival, compared with those with HCC of other aetiologies. Tailored surveillance strategies should be developed for patients with NAFLD without cirrhosis.

## Teaser

Patients with non-alcoholic fatty liver disease (NAFLD)-related hepatocellular carcinoma (HCC) have similar perioperative complications and mortality compared with those with HCC of other aetiologies. Patients with NAFLD-related HCC have potentially higher overall and recurrence-free survival than those with HCC of other aetiologies.

## Introduction

Hepatic resection is the only curative treatment for patients with hepatocellular carcinoma (HCC)^1–4^. The main causes of HCC are chronic alcohol abuse and chronic infection with hepatitis B virus (HBV) or hepatitis C virus (HCV). The incidence of viral hepatitis-related HCC has decreased in recent years with the emergence and popularization of anti-hepatitis vaccines and antiviral drugs^5,6^. Over the past decade, an increasingly prevalent risk factor for HCC in many countries is non-alcoholic fatty liver disease (NAFLD)^7,8^. NAFLD refers to a spectrum of liver damage ranging from simple steatosis to non-alcoholic steatohepatitis (NASH), then fibrosis and cirrhosis, and finally to hepatocarcinogenesis. Although the annual incidence of HCC is lower among NAFLD patients than among those infected with HBV or HCV, 25 per cent of the global population is estimated to have NAFLD—with even higher prevalence in high-income areas^9,10^—and the prevalence of NAFLD and NAFLD-related HCC is increasing in various countries^11–14^. These considerations highlight the importance of preventing and treating NAFLD-related HCC.

Increasing evidence indicates that the pathogenesis and clinical manifestations of NAFLD-related HCC differ from those of HCC with other aetiologies. In addition, diabetes mellitus and obesity promote the progression of NAFLD to HCC^15,16^. It is not clear whether these differences translate into a different prognosis after hepatic resection. Two meta-analyses have evaluated the safety and prognosis of hepatic resection for treating NAFLD-related HCC^17,18^, but they included relatively few original studies, and several relevant studies have been published since then^19–22^. Moreover, the meta-analyses were less rigorous in their inclusion criteria, such that some patients in the observation group did not meet diagnostic standards for NAFLD-related HCC^23–28^, and some patients received palliative treatment instead of resection^29–33^. Therefore, it was important to conduct an updated systematic review and meta-analysis of relevant clinical studies to compare prognosis after resection between patients with NAFLD-related HCC and patients with HCC of other aetiologies. This updated systematic review aims to provide a reference for selecting treatment for patients with NAFLD-related HCC.

## Methods

This systematic review and meta-analysis was performed according to the guidelines of MOOSE ('Meta-analysis Of Observational Studies in Epidemiology’)^34^ and PRISMA (*Appendix S1*). This was a systematic review pooling published data, so no ethical approval was required; since this study incorporated only previously published literature, approval from the local ethics committee to perform the research and informed consent from the patients in the included studies were not obtained.

### Inclusion and exclusion criteria

In this review, patients with NAFLD-related HCC were grouped together with patients whose HCC was related to metabolic-associated fatty liver disease (MAFLD), which is the term recently proposed to replace NAFLD. MAFLD has been defined as chronic disease involving metabolic dysfunction that leads to accumulation of fat in the liver, such that fat mass accounts for greater than 5 per cent of total liver mass^35^. Patients in the present review were diagnosed with MAFLD if they had hepatic steatosis as well as one of the following: overweight/obesity, defined as a body mass index (BMI) greater than or equal to 23 kg/m^2^; type 2 diabetes; or other evidence of metabolic disorder^35–38^. Although MAFLD is closely linked to these metabolic disorders^39^, its epidemiology is quite similar to that of NAFLD^40^, so we grouped together data for HCC patients with either NAFLD or MAFLD.

The inclusion criteria, structured according to participants, interventions, comparisons, and outcomes (PICO) were as follows: adult HCC patients without geographical or aetiological limitations; patients who underwent hepatic resection, including laparotomy or laparoscopic surgery; patients who could be assigned to either a group with HCC related to NAFLD or MAFLD, or a group with HCC of other aetiologies; studies reporting data on overall survival (OS) and/or recurrence-free survival (RFS) as outcomes; and studies with a cohort or case-control design. Randomized clinical trials were not included.

Exclusion criteria were as follows: the study was a case report, review, or meta-analysis; patients received other therapies for HCC, such as radiofrequency ablation, sorafenib, lenvatinib, or other first-line therapies; or patients had other malignancies in addition to HCC. When multiple studies involved overlapping patient populations, only the largest study was included.

### Data sources and literature search strategy

PubMed, EMBASE, Scopus, and Cochrane Library databases were systematically searched. Since NAFLD was first proposed in 1980, the database search time was set as 1 January 1980 to 25 June 2022, and the language of publication was limited to English. Search terms were: HCC, hepatoma, liver neoplasm, NAFLD, MAFLD, NASH, surgery, and survival. For example, the search string for the PubMed database was ‘(hepatocellular carcinoma OR liver neoplasm) AND (non-alcoholic fatty liver disease OR NAFLD OR nonalcoholic steatohepatitis OR NASH OR metabolic associated fatty liver disease OR MAFLD) AND (surgery OR resection OR hepatectomy) AND survival’.

### Literature screening and data extraction

Two researchers (J.-Y.S. and Z.-J.D.) independently searched and screened the literature, then extracted relevant data from the included studies. Inconsistencies in the data were resolved through discussion with a third researcher (J.-H.Z.). The literature was screened based on titles, abstracts, and, when necessary, the full text. The following data were extracted from included studies: name of first author, publication year, country, inclusion interval, sample size, HCC aetiology and rate in the control group, average or median age of subjects, BMI, rate of cirrhosis, tumour stage, perioperative mortality, incidence of complications, OS, and RFS. For raw data that were not available, efforts were made to contact the corresponding author.

### Outcomes

The primary outcome was the comparison of post-hepatectomy OS between patients with NAFLD- or MAFLD-related HCC (observation group) and patients with HCC with other aetiologies (control group). OS was defined as the time between the day of hepatectomy and the patient’s all-cause death or the end of follow-up. The secondary outcomes included RFS, perioperative mortality, and complication rates. RFS was defined as the time from the day of hepatectomy to the time of HCC recurrence or death, with the first HCC occurrence considered the primary event. Perioperative mortality was defined as death within 30 or 90 days after hepatectomy. Complications were defined as ‘major’ if they were graded as 3a or above according to the Clavien–Dindo classification system^41^. Other aetiologies of HCC included: viral hepatitis, related to HBV or HCV infection; alcoholic cirrhosis; and cryptogenic cirrhosis.

### Evaluation of study quality

Two researchers (J.-Y.S. and Z.-J.D.) independently evaluated the quality of the included studies using the Newcastle–Ottawa scale (NOS) for quality assessment (*Appendix S2*)^42^. The scale includes eight multiple-choice questions that relate to patient selection, comparisons, and outcomes. Studies were assigned up to nine stars; those receiving at least seven stars were considered of good quality, while those receiving four to six stars were considered of fair quality. Inconsistencies were resolved through discussion with a third researcher (J.-H.Z.).

### Statistical analysis

Continuous data were expressed as mean ± standard deviation if they showed a normal distribution, or as median (interquartile range) if they showed a skewed distribution. OS and RFS in both groups were assessed by combining log-transformed hazard ratios (HRs) and corresponding 95 per cent confidence intervals using an inverse variance, random-effects model. HRs and 95 per cent confidence intervals were extracted directly from the included literature; if these data were not reported, they were estimated indirectly using Parmar’s method^43^.

Statistical heterogeneity was assessed using Higgins’ *I*² statistic and Cochran’s Q test^44^. The *I*^2^ gives a percentage representing the magnitude of heterogeneity between groups. A percentage of 25 per cent was taken to indicate low heterogeneity, a percentage of 50 per cent was taken to indicate moderate heterogeneity, and a percentage of 75 per cent was taken to indicate high heterogeneity. Publication bias was assessed using Egger’s and Begg’s regression analysis of funnel plots^45^. Subgroup analysis was performed based on geographical region where studies were performed, or based on HCC aetiology. Sensitivity analysis was performed in which individual studies were omitted and the meta-analysis was repeated. Median OS and RFS at 1, 3, and 5 years were estimated with bubble charts, where the size of each bubble represented the sample size of the given study^3^. Meta-analyses were performed using RevMan (version 5.4.0) and R (version 3.5.0; R Foundation; http://cran.rproject.org). All statistical tests were two-sided, and differences associated with *P* < 0.050 were considered statistically significant.

## Results

### Literature search results

**Fig. 1:**
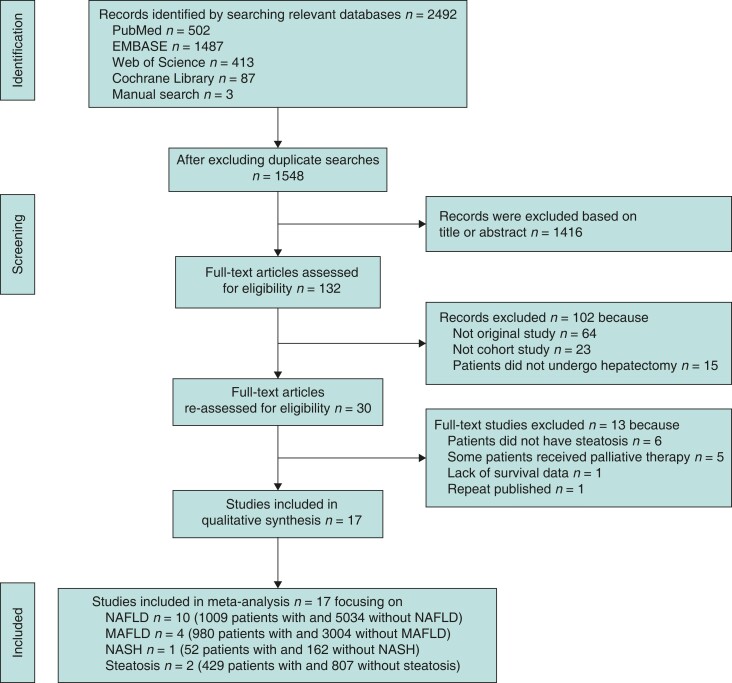
PRISMA diagram showing selection of articles for meta-analysis MAFLD, metabolic-associated fatty liver disease; NAFLD, non-alcoholic fatty liver disease; NASH, non-alcoholic steatohepatitis.

A total of 2492 publications were retrieved from relevant databases based on the inclusion and exclusion criteria. Thirteen publications were excluded because they included patients without steatosis^23–28^ or who received palliative treatment^29–33^, because the studies did not report survival data^46^ or because of repeat publication^47^. In the end, 17 retrospective studies were included (*Fig. 1*)^19–22,48–60^.

### Characteristics of included studies and patients

Basic characteristics of the literature included in this systematic review are shown in *Table 1*. The studies covered 28 years (1991–2019). Of the 17 studies, 10 applied standard definitions of NAFLD^21,48–51,54,55,57–59^, 4 applied standard definitions of MAFLD^19,20,22,60^, 2 applied standard definitions of steatosis^53,56^, and 1 applied a standard definition of NASH^52^. Patients in all studies except one underwent hepatectomy; those in the study applying a standard definition of NASH^52^ underwent liver transplantation, hepatectomy, and radiofrequency ablation, all of which are radical treatments. All studies had a retrospective cohort design and most (94.1 per cent) showed high quality except one with fair quality^53^ (*Table S1*).

**Table 1:** Baseline characteristics of patients with hepatocellular carcinoma associated with non-alcoholic fatty liver disease or metabolic-associated fatty liver disease and patients with hepatocellular carcinoma of other aetiologies

Study	Country/region and inclusion interval	Aetiology	Sample size	Age (years)*	BMI (kg/m^2^)*	Cirrhosis	Macrovascular invasion	Complications (Clavien–Dindo >3a)	90-day mortality
Liu 2022^19^	China, 2014–2018	MAFLD	67	58.8	≥23: 42 (62.7)	34 (50.7)	7 (10.4)	3 (4.5)	0 (0)
		MAFLD/HBV	176	49.4	≥23: 166 (94.3)	120 (68.2)	21 (11.9)	18 (10.2)	0 (0)
		HBV	1082	48.9	≥23: 360 (33.3)	771 (71.3)	197 (18.2)	85 (7.9)	2 (0.2)
Conci 2022^20^	Italy, 2008–2018	MAFLD	264	71	27.8	101 (38.3)	31 (11.7)	19 (7.2)	11 (4.2)
		HBV	205	68	24.0	122 (59.5)	53 (25.9)	17 (8.3)	5 (2.4)
		HCV	671	72	24.0	491 (73.2)	94 (14.0)	62 (9.2)	21 (3.1)
		Alcohol	175	70	25.0	115 (65.7)	14 (8.0)	11 (6.3)	3 (1.7)
Jung 2021^21^	South Korea, 2005–2015	NAFLD	32	61	26.6	5 (16)	1 (3.1)	2 (6)	2 (6)
		NAFLD/HBV	194	55	24.7	126 (65)	13 (6.7)	15 (8)	3 (2)
		HBV	200	54	23.2	110 (55)	14 (7.0)	11 (6)	5 (3)
Lin 2021^22^	Taiwan, 2010–2019	MAFLD/HBV	369	56.2	26.7	181 (49.1)	0 (0)	–	–
		HBV	443	56.2	23.9	229 (51.7)	0 (0)	–	–
Kimura 2017^48^	Japan, 1996–2012	NAFLD	30	71	25.0	8 (27)	7 (23.3)	–	–
		Alcohol	31	69	22.4	18 (58)	11 (35.5)	–	–
		Cryptogenic	16	75	22.6	4 (25)	12 (75.0)	–	–
Koh 2019^49^	Singapore, 2000–2015	NAFLD	152	69	25.4	52 (34.2)	–	24 (16.2)	3 (1.99)
		Non-NAFLD	844	63	25.0	429 (51.1)	–	68 (8.1)	21 (2.46)
Mikuriya 2015^50^	Japan, 1998–2011	NAFLD	21	69.3	–	–	–	–	–
		HCV	645	66.3	–	–	–	–	–
Pais 2017^51^	France, 1995–2014	NAFLD	39	70	27.6	9 (23.1)	17 (43.6)	–	–
		Non-NAFLD	284	58	24.4	182 (64.1)	122 (43.0)	–	–
Reddy 2012^52^	USA, 2000–2010	NASH	52	65	31.3	–	3 (5.8)	–	–
		HCV/alcohol	162	58	28.7	–	14 (8.6)	–	–
Su 2015^53^	Taiwan, 1991–2006	Steatosis	74	60.5	25.1	43 (58.1)	3 (4.1)	–	–
		No steatosis	114	62.5	23.0	68 (59.6)	4 (3.5)	–	–
Wakai 2011^54^	Japan, 1990–2007	NAFLD	17	>65: 13 (76.5)	>25: 11 (64.7)	9 (52.9)	7 (41.2)	All: 10 (58.8)	2 (11.8)
		HBV	61	>65: 14 (23.0)	>25: 12 (19.7)	39 (63.9)	27 (44.3)	All: 17 (27.9)	2 (3.3)
		HCV	147	>65: 84 (57.1)	>25: 24 (16.3)	84 (57.1)	41 (27.9)	All: 45 (30.6)	1 (0.7)
Wong 2017^55^	USA, 1991–2011	NAFLD	178	–	–	–	–	–	–
		HCV	413	–	–	–	–	–	–
		HBV	215	–	–	–	–	–	–
		Alcohol	60	–	–	–	–	–	–
Wu 2011^56^	Taiwan, 1999–2005	Steatosis	355	57.5	25.2	208 (58.6)	111 (31.3)	All: 106 (29.9)	16 (4.5)
		No steatosis	693	55.7	23.6	396 (57.1)	277 (40.0)	All: 192 (27.7)	34 (4.9)
Yang 2020^57^	China, 2003–2014	NAFLD	96	57.3	-	29 (30.2)	10 (10.4)	8 (9.4)	1 (1.0)
		HBV	1387	50.0	-	1006 (72.5)	195 (14.1)	140 (10.1)	19 (1.4)
Yoon 2020^58^	South Korea, 2009–2013	NAFLD/HBV	196	55.0	24.1	85 (43.4)	–	–	–
		HBV	142	57.0	22.5	54 (38.0)	–	–	–
D'Silva 2022^59^	South Korea, 2004–2018	NAFLD	54	65.8	>25: 38 (70.4)	33 (61.1)	–	4 (7.4)	–
		HBV	589	56.4	>25: 239 (40.6)	364 (61.8)	–	72 (12.2)	–
Xiong 2022^60^	China, 2015–2018	MAFLD/HBV	104	57.0	24.5	76 (73.1)	0 (0)	–	–
		HBV	428	57.0	22.2	324 (75.7)	0 (0)	–	–

Values are *n* (%) unless otherwise indicated. *Median or mean. BMI, body mass index; MAFLD, metabolic-associated fatty liver disease; HBV, hepatitis B virus; HCV, hepatitis C virus; Alcohol, alcoholic cirrhosis; NAFLD, non-alcoholic fatty liver disease; Cryptogenic, cryptogenic cirrhosis.

**Fig. 2:**
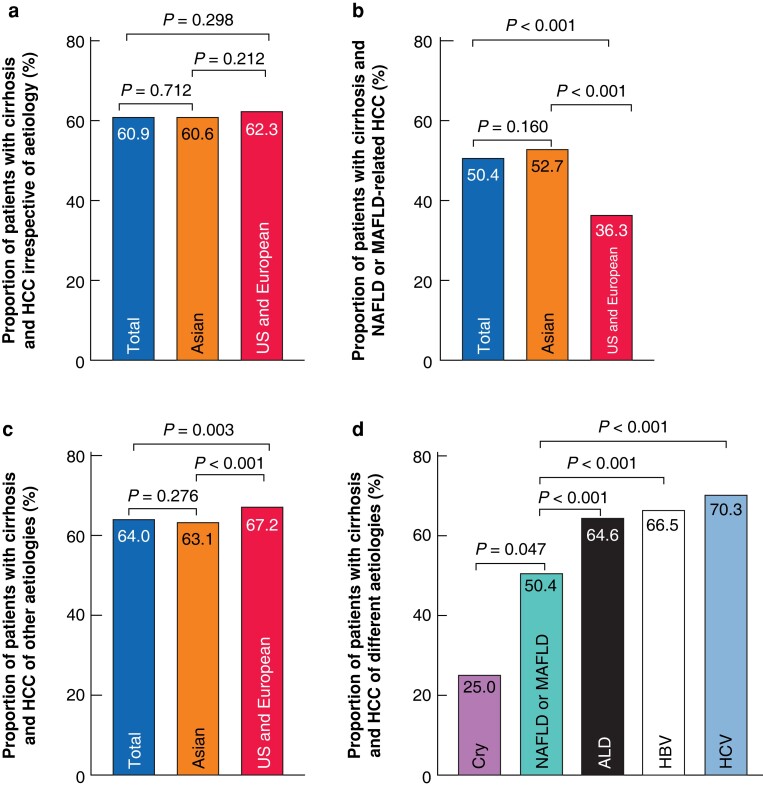
Proportion of patients with cirrhosis and hepatocellular carcinoma **a** HCC irrespective of aetiology. **b** HCC related to NAFLD or MAFLD. **c** HCC of other aetiologies. **d** HCC related to different aetiologies. ALD, alcoholic cirrhosis disease; Cry, cryptogenic; HBV, hepatitis B virus; HCC, hepatocellular carcinoma; HCV, hepatitis C virus; MAFLD, metabolic-associated fatty liver disease; NAFLD, non-alcoholic fatty liver disease.

The 17 studies included 11 477 patients, including 2470 (21.5 per cent) with NAFLD-related HCC and 9007 (78.5 per cent) with HCC of other aetiologies (*Table 1*). The prevalence of NALFD-related HCC in Asian patients was significantly higher compared with that in European and US patients (*P* = 0.006; *Fig. S1*). Compared with patients with HCC of other aetiologies, those with NAFLD-related HCC had a higher BMI, they were older in 11 studies^19,21,49–52,54–57,59^, and they showed significantly lower cirrhosis rates in 11 studies^19–22,48,49,51,53–55,57^ of the 15 that reported cirrhosis rates^19–22,48,49,51,53–60^. When cirrhosis rates were calculated across all patients pooled from the studies, prevalence was significantly lower in the observation group than in the control group (*P* < 0.001). Patients with HCC irrespective of aetiology from Asia and those from the USA and Europe had a similar proportion of cirrhosis (*P* = 0.212; *Fig. 2a*). Among patients with NAFLD-related HCC, patients from Asia had a significantly higher proportion of cirrhosis than those from the USA and Europe (*P* < 0.001; *Fig. 2b*). However, among patients with HCC of other aetiologies, US and European patients had a significantly higher proportion of cirrhosis than those from Asia (*P* < 0.001; *Fig. 2c*). Moreover, the proportion of cirrhosis among patients with NAFLD-related HCC was significantly lower than that among those with alcohol-, HBV-, or HCV-related HCC (all *P* < 0.001; *Fig. 2d*).

### Perioperative complications and mortality

Eight studies reported the incidence of perioperative complications, which was higher in the observation group than in the control group in four studies^49,54,56,59^, lower in the observation group in two studies^19,57^, and similar between the observation and control groups in the other two studies^20,21^. Pooling the patients across all eight studies showed that the rate of major complications was similar between the observation group (8.98 per cent, 93/1035) and control group (9.0 per cent, 466/5153; *P* = 1.000).

Seven studies reported perioperative mortality, which was higher in the observation group in three studies^20,21,54^, but similar between the observation and control groups in the remaining four studies^19,49,56,57^. When rates of perioperative mortality were calculated across all patients pooled from the studies, the rate was similar between the observation group (2.9 per cent, 35/1201) and control group (2.1 per cent, 116/5617; *P* = 0.083).

### Overall survival

**Fig. 3:**
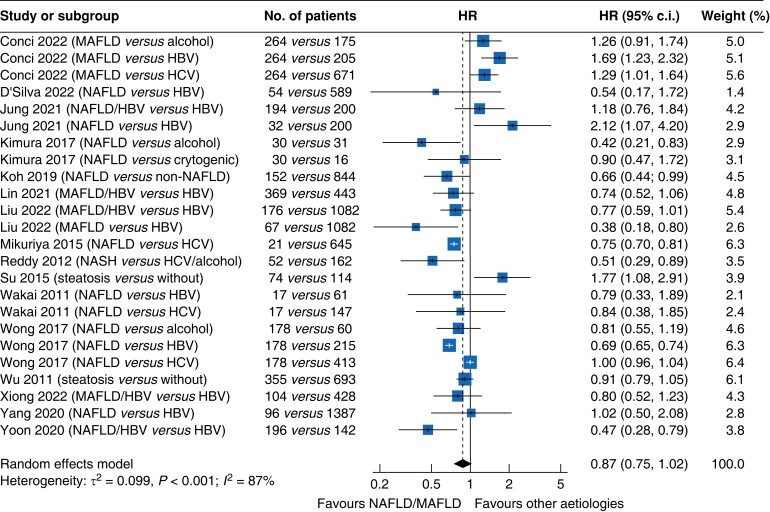
Comparison of overall survival between patients with hepatocellular carcinoma related to non-alcoholic fatty liver disease or metabolic-associated fatty liver disease and patients with hepatocellular carcinoma of other aetiologies HBV, hepatitis B virus; HCV, hepatitis C virus; MAFLD, metabolic-associated fatty liver disease; NAFLD, non-alcoholic fatty liver disease; NASH, non-alcoholic steatohepatitis.

**Fig. 4:**
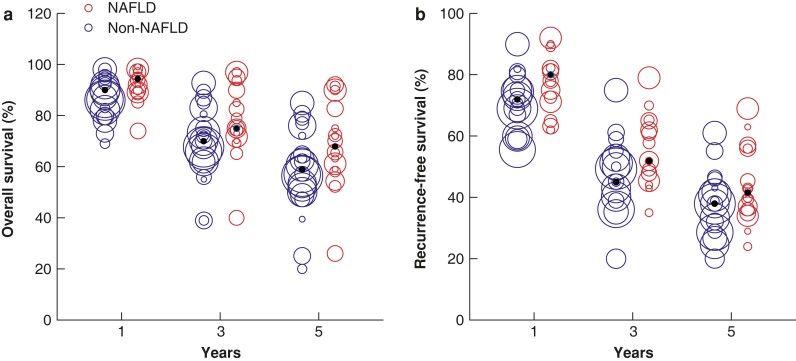
Bubble plots of 1-, 3-, and 5-year survival of patients with hepatocellular carcinoma related to non-alcoholic fatty liver disease or metabolic-associated fatty liver disease and patients with hepatocellular carcinoma of other aetiologies **a** Overall survival. **b** Recurrence-free survival. The size of each bubble represents the sample size of the given study NAFLD, non-alcoholic fatty liver disease.

Data pooled from the 16 studies that reported OS^19–22,48–50,52–60^ showed that patients with NAFLD-related HCC had marginally higher OS than those with HCC of other aetiologies (HR 0.87, 95 per cent c.i. 0.75 to 1.02, *P* = 0.051; *Fig. 3*), as well as higher median OS rates at 1 year (94.5 per cent *versus* 90.0 per cent), 3 years (75.3 per cent *versus* 70.2 per cent), and 5 years (67.9 per cent *versus* 59.0 per cent; *Fig. 4a*). This difference became significant among the subset of studies involving Asian patients (*P* < 0.001; *Fig. S2*), but not among the subset of studies involving patients from the US and Europe (*P* = 0.934; *Fig. S2*).

In subgroup analyses based on HCC aetiology, patients with NAFLD-related HCC showed similar OS to those with HCC related to HBV or HCV infection (*P* = 0.134; *Fig. S3*) and those with HCC related to alcoholic or cryptogenic cirrhosis (*P* = 0.413; *Fig. S3*). In contrast, the OS of patients with HCC related specifically to NAFLD, based on studies applying standard definitions of NAFLD^21,48–51,54,55,57–59^, was significantly better than the OS of patients with HCC of other aetiologies (*P* = 0.012; *Fig. S4*).

### Recurrence-free survival

**Fig. 5:**
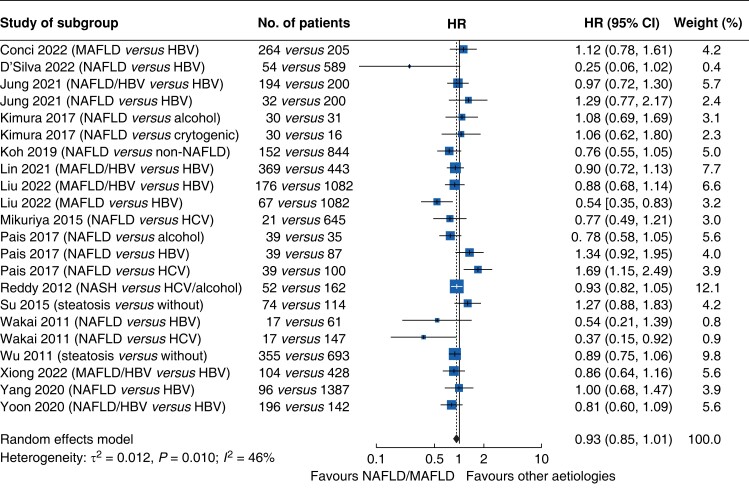
Comparison of recurrence-free survival between patients with hepatocellular carcinoma related to non-alcoholic fatty liver disease or metabolic-associated fatty liver disease and patients with hepatocellular carcinoma of other aetiologies HBV, hepatitis B virus; HCV, hepatitis C virus; MAFLD, metabolic-associated fatty liver disease; NAFLD, non-alcoholic fatty liver disease; NASH, non-alcoholic steatohepatitis.

Data pooled from the 16 studies that reported RFS^19–22,48–54,56–60^ showed that patients with NAFLD-related HCC had marginally better RFS than patients with HCC of other aetiologies (HR 0.93, 95 per cent c.i. 0.85 to 1.01, *P* = 0.128; *Fig. 5*), as well as higher median RFS rates at 1 year (80.0 per cent *versus* 72.0 per cent), 3 years (52.1 per cent *versus* 45.3 per cent), and 5 years (41.6 per cent *versus* 38.0 per cent; *Fig. 4b*). This difference became significant among the subset of studies involving Asian patients (*P* = 0.02; *Fig. S5*), but not among the subset of studies involving patients from the US and Europe (*P* = 0.466; *Fig. S5*).

In subgroup analyses based on HCC aetiology, patients with NAFLD-related HCC showed similar RFS to those with HCC related to HBV or HCV infection (*P* = 0.283; *Fig. S6*) and those with HCC related to alcoholic or cryptogenic cirrhosis (*P* = 0.327; *Fig. S6*). A similar result was found when considering the RFS of patients with HCC related specifically to NAFLD, based on studies applying standard definitions of NAFLD^21,48–51,54,55,57–59^ (*P* = 0.464; *Fig. S7*).

### Sensitivity analysis

None of the meta-analysis findings changed after the exclusion of patients who underwent liver transplantation or radiofrequency ablation^52^. Excluding each study one by one altered the meta-analysis of OS in the case of Conci 2022^20^ (MAFLD *versus* HBV), Jung 2021^21^ (MAFLD *versus* HBV), and Su 2015^53^ (steatosis *versus* without) (*Fig. S8*). Moreover, excluding each study one by one altered the meta-analysis of RFS in the case of Liu 2022^19^ (MAFLD *versus* HBV), Pais 2017^51^ (NAFLD *versus* HBV), Pais 2017^51^ (NAFLD *versus* HCV), and Su 2015^53^ (steatosis *versus* without) (*Fig. S8*).

### Publication bias

No significant publication bias was found in funnel plots for OS (*P* = 0.487; *Fig. S9*) or RFS (*P* = 0.672; *Fig. S9*).

## Discussion

As NAFLD/MAFLD becomes more prevalent, treating HCC related to NAFLD/MAFLD becomes relevant. In this context, it may help clinicians and patients in their treatment decisions to know whether post-hepatectomy outcomes differ significantly between patients with NAFLD- or MAFLD-related HCC and patients with HCC of other aetiologies. The present meta-analysis of 11 477 cases from 17 studies suggests that the two groups of patients show similar rates of major perioperative complications, OS, and RFS after hepatic resection. However, when focusing on studies performed in Asia, significantly better OS and RFS for patients with NAFLD- or MAFLD-related HCC than for those with HCC of other aetiologies were found.

The finding of similar OS and RFS between the two groups of patients contrasts with the significantly higher survival reported for patients with NAFLD- or MAFLD-related HCC in two previous meta-analyses involving 7226^17^ and 5579 patients respectively^18^. The present results may be more reliable because the sample is substantially larger (11 477 patients) and the inclusion criteria are more stringent. All patients in the analysis underwent hepatectomy, and all patients in the observation group fulfilled standard diagnostic criteria for NAFLD- or MAFLD-related HCC. Nevertheless, the meta-analyses showed substantial statistical heterogeneity, especially the meta-analysis of OS. This may reflect that patients were enrolled over a 28-year interval, different medical centres may define fatty liver in different ways, and the patient samples in individual studies differed substantially in the rates of cirrhosis. The influence of these factors on the meta-analyses could not be assessed because of the lack of patient-level data.

The patients with NAFLD- or MAFLD-related HCC in this review were older and more likely to have diabetes, obesity, hyperlipidaemia, or other metabolic disorders than the patients with HCC of other aetiologies. Nevertheless, the two groups showed similar rates of major perioperative complications (8.98 per cent *versus* 9.0 per cent, *P* = 1.000) and mortality (2.9 per cent *versus* 2.1 per cent, *P* = 0.083). These results likely reflect the substantial improvement in surgical techniques and perioperative management of hepatectomy. A systematic review of 14 808 patients who underwent hepatectomy for intermediate or advanced HCC found a low perioperative mortality of 2.7 per cent^61^.

One explanation for the finding, across all studies, that patients with NAFLD- or MAFLD-related HCC have slightly better OS or RFS than patients with HCC of other aetiologies is the fact that the first group of patients showed a significantly lower prevalence of cirrhosis (50.4 per cent *versus* 64.0 per cent, *P* < 0.001). Cirrhosis is an independent risk factor for all-cause mortality in patients with NAFLD, NASH, or HCC. The finding that OS and RFS were significantly higher among patients with NAFLD- or MAFLD-related HCC than among those with HCC of other aetiologies in Asia but not in Europe and the USA may reflect regional differences in HCC aetiology and tumour characteristics^62,63^. For example, hepatectomy is often used in Asia to treat HCC patients for whom surgery is not recommended in the USA or Europe^2,64–66^. Finally, the results may also reflect the relatively small number of patients from Europe and the USA in the meta-analysis.

Many of the patients in five studies^29–33^ of a previous meta-analysis^17^ not included in the present one received transarterial chemoembolization, radiotherapy, chemotherapy, or best supportive care instead of hepatectomy. All five of those studies reported similar OS between patients with NAFLD- or NASH-related HCC and patients with HCC related to viral hepatitis or alcohol (*Table S2*). However, other studies found that lenvatinib treatment may lead to better OS, while immune checkpoint inhibitors may lead to worse OS when treating NAFLD-related HCC compared with HCC of other aetiologies^67,68^. In addition to hepatectomy, liver transplantation and radiofrequency ablation can be applied as radical therapies against HCC. One meta-analysis reported similar post-transplantation OS between patients with NASH-related HCC or HCC of other aetiologies^69^. Two cohort studies found similar post-ablation OS between patients with NAFLD-related HCC and patients with HCC of other aetiologies^55,70^.

Half of the patients with NAFLD-related HCC had no cirrhosis. This rate was as high as 63.7 per cent in the US and European populations. However, the proportion of patients without cirrhosis among those with alcohol-, HBV-, or HCV-related HCC was only 35.4 per cent, 33.5 per cent, and 29.7 per cent respectively. Therefore, patients with NAFLD without cirrhosis still need regular surveillance for HCC. This finding based on patients with HCC after hepatectomy was consistent with a previous meta-analysis that included patients with NAFLD-related HCC who underwent curative and palliative treatment strategies^71^.

Despite the stricter inclusion criteria, the present meta-analysis involved a substantially larger sample than previous ones. In addition, the present meta-analysis included MAFLD-related HCC and took into account the prevalence of cirrhosis, incidence of major perioperative complications, and mortality. However, it was limited by the fact that the included studies had a retrospective cohort design, with no access to patient-level data. Most patients in the meta-analysis came from Asia, which may reduce the generalizability of the results to other regions. Finally, heterogeneity among included studies was large.

This study systematically reviewed post-hepatectomy outcomes of patients with HCC of different aetiologies. The analysis suggests that patients with NAFLD- or MAFLD-related HCC show similar major perioperative complications, mortality, OS, and RFS compared with patients with HCC of other aetiologies. However, Asian patients may have better survival if their HCC is related to NAFLD or MAFLD. The surveillance for HCC in NAFLD patients without cirrhosis is equally important as surveillance for HCC in NAFLD patients with cirrhosis.

## Supplementary Material

zrac167_Supplementary_Data

## Data Availability

All data are in the manuscript. Supplementary material for this article is available online.
