# Supplementary material for: Prognosis after hepatic resection of patients with hepatocellular carcinoma related to non-alcoholic fatty liver disease: meta-analysis
Source: BJS Open. 2023 Feb 20;7(1):zrac167. doi: 10.1093/bjsopen/zrac167 (PMC9939291; doi:10.1093/bjsopen/zrac167)
Supplement: zrac167_Supplementary_Data [file zrac167_supplementary_data.docx]

**Title: Prognosis after hepatic resection of patients with hepatocellular carcinoma related to non-alcoholic fatty liver disease: meta-analysis**

*Jia-Yong Su^1^, Zhu-Jian Deng^1^, Yu-Xian Teng^1^, Ye Xin Koh^2^, Wan-Guang Zhang^3^, Ming-Hua Zheng^4^, Si Xie^1^, Rong-Rui Huo^1^, Chao-Jing Chen^1^, Liang Ma^1^, Jian-Hong Zhong^1,5*^*

1. *Hepatobiliary Surgery Department, Guangxi Medical University Cancer Hospital, Nanning, China*
2. *Department of Hepatopancreatobiliary and Transplant Surgery, Singapore General Hospital, Singapore*
3. *Hepatic Surgery Center, Tongji Hospital, Tongji Medical College, Huazhong University of Science and Technology, Wuhan, China*
4. *NAFLD Research Center, Department of Hepatology, the First Affiliated Hospital of Wenzhou Medical University, Wenzhou, China*
5. *Key Laboratory of Early Prevention and Treatment for Regional High Frequency Tumor (Guangxi Medical University), Ministry of Education; Guangxi Key Laboratory of Early Prevention and Treatment for Regional High Frequency Tumor*

******Correspondence author****: Jian-Hong Zhong (钟鉴宏), Hepatobiliary Surgery Department, Guangxi Medical University Cancer Hospital, He Di Rd. #71, Nanning 530021, P.R. China. Tel: +86-771-5330855 (office). Fax: +86-771-5312000. Email:* [*zhongjianhong@gxmu.edu.cn*](mailto:zhongjianhong@gxmu.edu.cn)*.* **ORCID: 0000-0002-1494-6396**

**Supplementary Materials - Index**

| **Supplementary Appendixes** |  |
| --- | --- |
| Appendix S1. PRISMA-2009-checklist | *pag. 3* |
| Appendix S2. NOS quality assessment form | *pag. 5* |
| **Supplementary Figures and Tables** |  |
| Figure S1. Proportion of patients with hepatocellular carcinoma related to non-alcoholic fatty liver disease (NAFLD) or metabolic-associated fatty liver disease (MAFLD). | *pag. 6* |
| Figure S2. Comparison of overall survival between patients with hepatocellular carcinoma (HCC) related to non-alcoholic fatty liver disease (NAFLD) or metabolic-associated fatty liver disease (MAFLD), and those with HCC of other etiologies among the subsets of (up) Asian patients or (down) US and European patients. | *pag.7* |
| Figure S3. Comparison of overall survival between patients with hepatocellular carcinoma (HCC) related to non-alcoholic fatty liver disease (NAFLD) or metabolic-associated fatty liver disease (MAFLD), and those with HCC of other etiologies among the subsets of (up) HBV or HCV related HCC or (down) those with HCC related to alcoholic or cryptogenic cirrhosis. | *pag.8* |
| Figure S4. Comparison of overall survival between patients with hepatocellular carcinoma (HCC) related to non-alcoholic fatty liver disease (NAFLD), and those with HCC of other etiologies. | *pag.9* |
| Figure S5. Comparison of recurrence-free survival between patients with hepatocellular carcinoma (HCC) related to non-alcoholic fatty liver disease (NAFLD) or metabolic-associated fatty liver disease (MAFLD), and those with HCC of other etiologies among the subsets of (up) Asian patients or (down) US and European patients. | *pag.10* |
| Figure S6. Comparison of recurrence-free survival between patients with hepatocellular carcinoma (HCC) related to non-alcoholic fatty liver disease (NAFLD) or metabolic-associated fatty liver disease (MAFLD), and those with HCC of other etiologies among the subsets of (up) HBV or HCV related HCC or (down) those with HCC related to alcoholic or cryptogenic cirrhosis. | *pag.11* |
| Figure S7. Comparison of recurrence-free survival between patients with hepatocellular carcinoma (HCC) related to non-alcoholic fatty liver disease (NAFLD), and those with HCC of other etiologies. | *pag.12* |
| Figure S8. Sensitivity analysis (“leave-one-out”) on the meta-analysis of (up) overall survival or (down) recurrence-free survival in the entire cohort. | *pag.13* |
| Figure S9. Publication bias in the meta-analysis of (up) overall survival or (down) recurrence-free survival in the entire cohort, as estimated by Begg’s test. | *pag.14* |
| Table S1: QA assessment result. | *pag. 15* |
| Table S2. Comparison of prognosis of patients with NAFLD-related HCC or HCC of other etiologies (only some cases received hepatectomy). | *pag. 16* |
|  |  |

**Supplementary Appendixes**

**Appendix S1. PRISMA 2009 Checklist.**

| **Section/topic** | **#** | **Checklist item** | **Reported on page #** |
| --- | --- | --- | --- |
| **TITLE** | | |  |
| Title | 1 | Identify the report as a systematic review, meta-analysis, or both. | 1 |
| **ABSTRACT** | | |  |
| Structured summary | 2 | Provide a structured summary including, as applicable: background; objectives; data sources; study eligibility criteria, participants, and interventions; study appraisal and synthesis methods; results; limitations; conclusions and implications of key findings; systematic review registration number. | 2 |
| **INTRODUCTION** | | |  |
| Rationale | 3 | Describe the rationale for the review in the context of what is already known. | 3 |
| Objectives | 4 | Provide an explicit statement of questions being addressed with reference to participants, interventions, comparisons, outcomes, and study design (PICOS). | 4 |
| **METHODS** | | |  |
| Protocol and registration | 5 | Indicate if a review protocol exists, if and where it can be accessed (e.g., Web address), and, if available, provide registration information including registration number. | NA |
| Eligibility criteria | 6 | Specify study characteristics (e.g., PICOS, length of follow-up) and report characteristics (e.g., years considered, language, publication status) used as criteria for eligibility, giving rationale. | 4-5 |
| Information sources | 7 | Describe all information sources (e.g., databases with dates of coverage, contact with study authors to identify additional studies) in the search and date last searched. | 5 |
| Search | 8 | Present full electronic search strategy for at least one database, including any limits used, such that it could be repeated. | 5 |
| Study selection | 9 | State the process for selecting studies (i.e., screening, eligibility, included in systematic review, and, if applicable, included in the meta-analysis). | 5 |
| Data collection process | 10 | Describe method of data extraction from reports (e.g., piloted forms, independently, in duplicate) and any processes for obtaining and confirming data from investigators. | 5-6 |
| Data items | 11 | List and define all variables for which data were sought (e.g., PICOS, funding sources) and any assumptions and simplifications made. | 6 |
| Risk of bias in individual studies | 12 | Describe methods used for assessing risk of bias of individual studies (including specification of whether this was done at the study or outcome level), and how this information is to be used in any data synthesis. | 6 |
| Summary measures | 13 | State the principal summary measures (e.g., risk ratio, difference in means). | 7 |
| Synthesis of results | 14 | Describe the methods of handling data and combining results of studies, if done, including measures of consistency (e.g., I^2^) for each meta-analysis. | 7 |

Page 1 of 2

| **Section/topic** | **#** | **Checklist item** | **Reported on page #** |
| --- | --- | --- | --- |
| Risk of bias across studies | 15 | Specify any assessment of risk of bias that may affect the cumulative evidence (e.g., publication bias, selective reporting within studies). | 7 |
| Additional analyses | 16 | Describe methods of additional analyses (e.g., sensitivity or subgroup analyses, meta-regression), if done, indicating which were pre-specified. | 7 |
| **RESULTS** | | |  |
| Study selection | 17 | Give numbers of studies screened, assessed for eligibility, and included in the review, with reasons for exclusions at each stage, ideally with a flow diagram. | 8 |
| Study characteristics | 18 | For each study, present characteristics for which data were extracted (e.g., study size, PICOS, follow-up period) and provide the citations. | 8 |
| Risk of bias within studies | 19 | Present data on risk of bias of each study and, if available, any outcome level assessment (see item 12). | 9 |
| Results of individual studies | 20 | For all outcomes considered (benefits or harms), present, for each study: (a) simple summary data for each intervention group (b) effect estimates and confidence intervals, ideally with a forest plot. | 9 |
| Synthesis of results | 21 | Present results of each meta-analysis done, including confidence intervals and measures of consistency. | 9-11 |
| Risk of bias across studies | 22 | Present results of any assessment of risk of bias across studies (see Item 15). | 10 |
| Additional analysis | 23 | Give results of additional analyses, if done (e.g., sensitivity or subgroup analyses, meta-regression [see Item 16]). | 11 |
| **DISCUSSION** | | |  |
| Summary of evidence | 24 | Summarize the main findings including the strength of evidence for each main outcome; consider their relevance to key groups (e.g., healthcare providers, users, and policy makers). | 12 |
| Limitations | 25 | Discuss limitations at study and outcome level (e.g., risk of bias), and at review-level (e.g., incomplete retrieval of identified research, reporting bias). | 12-13 |
| Conclusions | 26 | Provide a general interpretation of the results in the context of other evidence, and implications for future research. | 13-14 |
| **FUNDING** | | |  |
| Funding | 27 | Describe sources of funding for the systematic review and other support (e.g., supply of data); role of funders for the systematic review. | 14 |

*From:*  Moher D, Liberati A, Tetzlaff J, Altman DG, The PRISMA Group (2009). Preferred Reporting Items for Systematic Reviews and Meta-Analyses: The PRISMA Statement. PLoS Med 6(7): e1000097. doi:10.1371/journal.pmed1000097

For more information, visit: **www.prisma-statement.org**.

**Supplementary Appendixes**

**Appendix S2. NOS quality assessment form.**

**NAFLD/MAFLD-related HCC MA QA Form**

**NEWCASTLE - OTTAWA QUALITY ASSESSMENT SCALE for COHORT STUDIES**

**Reviewer: Review date:**

**Study Title:**

Note: A study can be awarded a maximum of one star for each numbered item within the Selection and Outcome categories. A maximum of two stars can be given for Comparability

**Selection: (Score:___) (Maximum: 4)**

1) Representativeness of the NAFLD/MAFLD cohort

a) truly representative of the average NAFLD or MAFLD population in the community in multiple centers with large sample size (at least 100 in each arm)****

b) multiple centers or sample size >100 in either arm****

c) single center and sample size <100 in either arm

d) no description of the derivation of the cohort

2) Selection of the HCC of other etiologies cohort

a) drawn from the same community as the NAFLD/MAFLD cohort over similar time period or follow up ****

b) drawn from a different source or different time

c) no description of the derivation of the cohort

3) Ascertainment of exposure

a) independent assess (the diagnoses of NAFLD/MAFLD were confirmed by preoperative imaging and/or postoperative histopathology) ****

b) electronic exhibition without validation

c) written self report

d) no description

4) Demonstration that outcome of interest was not present at start of study

a) yes (death and tumour recurrence occur after hepatectomy)****

b) no

**Comparability: (Score:___) (Maximum: 2)**

1) Comparability of cohorts on the basis of the design or analysis

a) propensity score matching was performed ****

b) unmatched studies but analysis control for age, gender, cirrhosis, and tumour stage ****

c) single arm study

**Outcome: (Score:___) (Maximum: 3)**

1) Assessment and follow-up of outcome

a) independent assessment ****

b) record linkage ****

c) self report

d) no description

2) Was median follow-up at least 3 years

a) yes ****

b) no

3) Was median follow-up >3 years for at least 80% of the cohort

a) yes****

b) no

**Supplementary Figures and Tables**

**
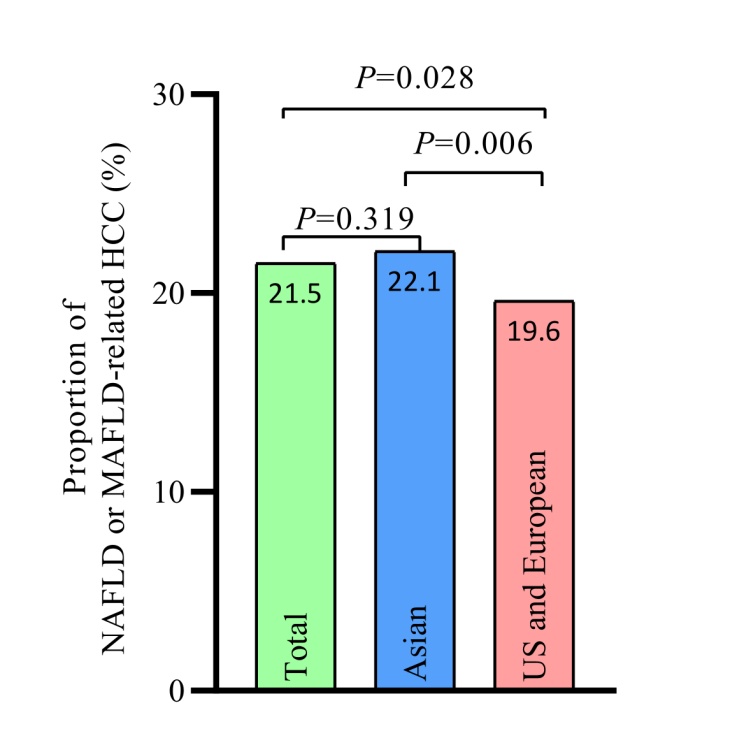
**

**Figure S1.** Proportion of patients with hepatocellular carcinoma related to non-alcoholic fatty liver disease (NAFLD) or metabolic-associated fatty liver disease (MAFLD).

**
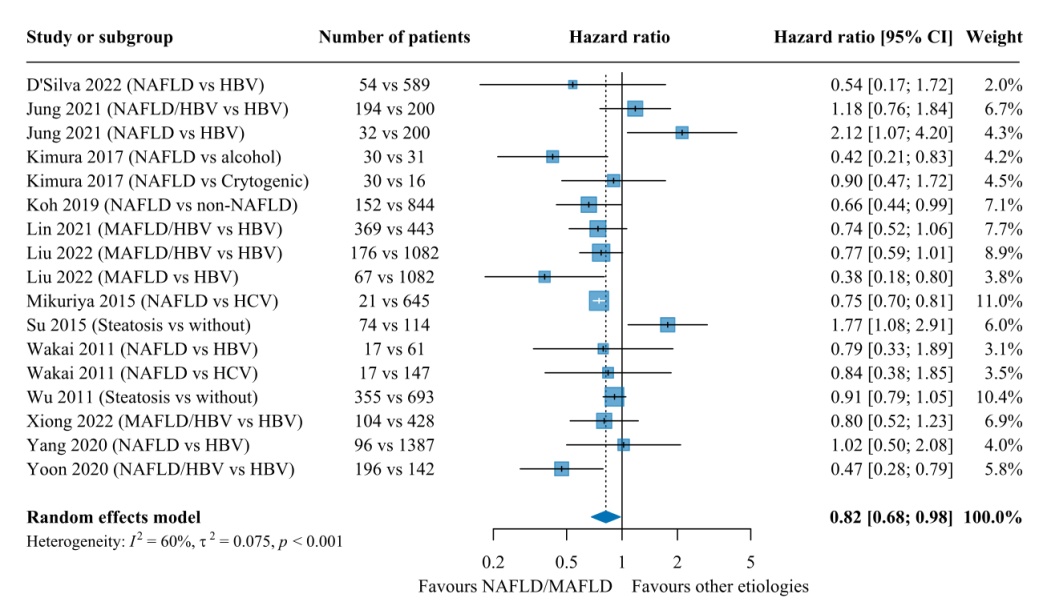
**

**
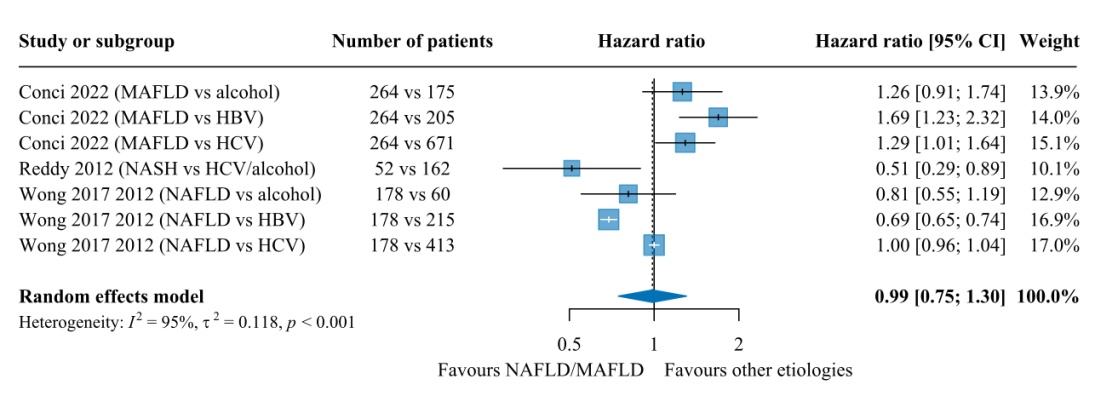
**

**Figure S2.** Comparison of overall survival between patients with hepatocellular carcinoma (HCC) related to non-alcoholic fatty liver disease (NAFLD) or metabolic-associated fatty liver disease (MAFLD), and those with HCC of other etiologies among the subsets of (up) Asian patients or (down) US and European patients.

HBV, hepatitis B virus; HCV, hepatitis C virus; MAFLD, metabolic-associated fatty liver disease; NAFLD, non-alcoholic fatty liver disease; NASH, nonalcoholic steatohepatitis.


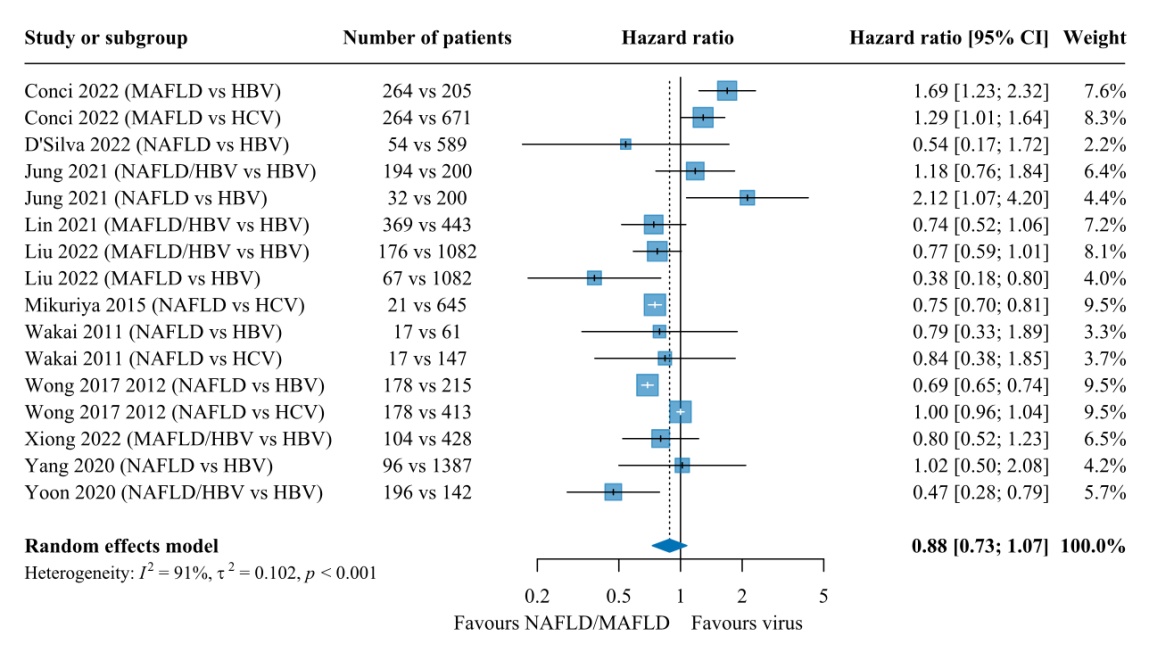

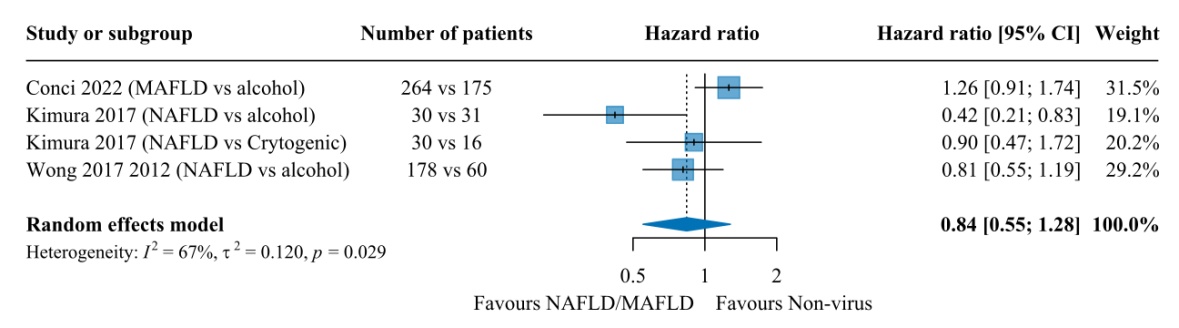


**Figure S3.** Comparison of overall survival between patients with hepatocellular carcinoma (HCC) related to non-alcoholic fatty liver disease (NAFLD) or metabolic-associated fatty liver disease (MAFLD), and those with HCC of other etiologies among the subsets of (up) HBV or HCV related HCC or (down) those with HCC related to alcoholic or cryptogenic cirrhosis.

HBV, hepatitis B virus; HCV, hepatitis C virus; NASH, nonalcoholic steatohepatitis.


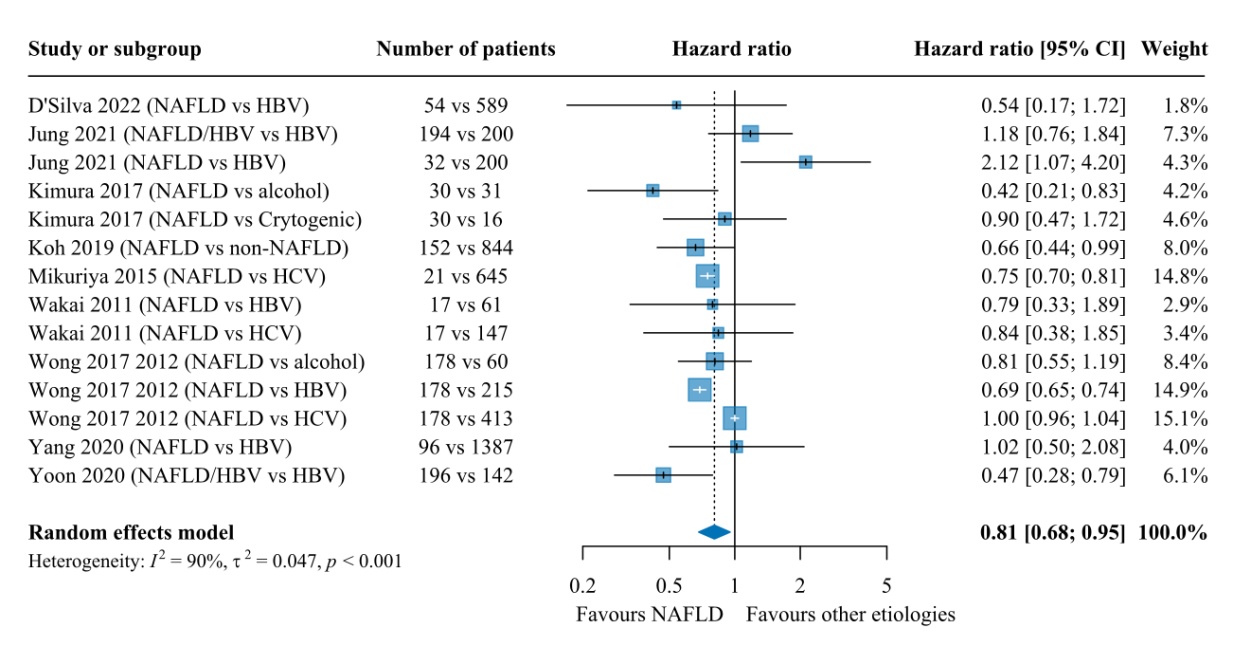


**Figure S4.** Comparison of overall survival between patients with hepatocellular carcinoma (HCC) related to non-alcoholic fatty liver disease (NAFLD), and those with HCC of other etiologies.

HBV, hepatitis B virus; HCV, hepatitis C virus.


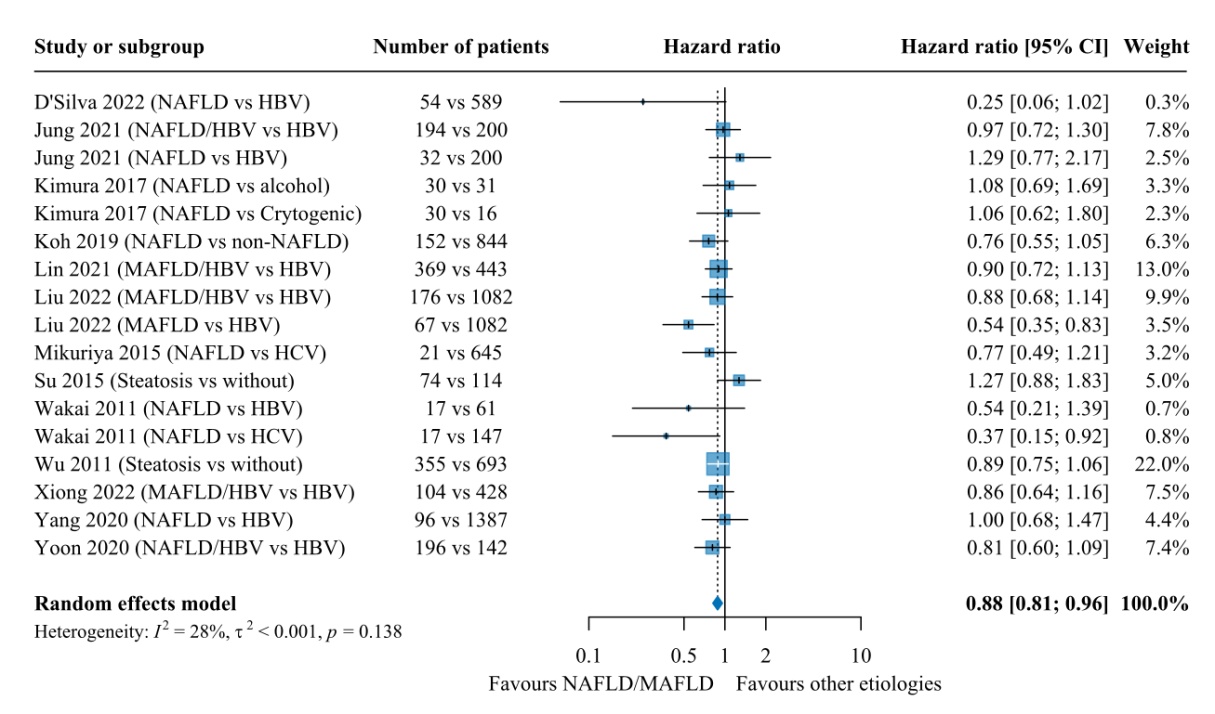


**
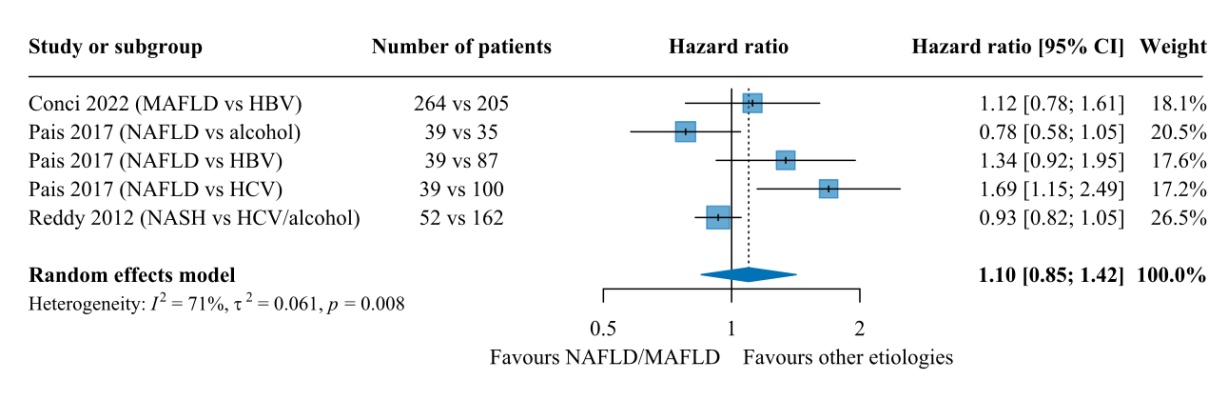
**

**Figure S5.** Comparison of recurrence-free survival between patients with hepatocellular carcinoma (HCC) related to non-alcoholic fatty liver disease (NAFLD) or metabolic-associated fatty liver disease (MAFLD), and those with HCC of other etiologies among the subsets of (up) Asian patients or (down) US and European patients.

HBV, hepatitis B virus; HCV, hepatitis C virus; NASH, nonalcoholic steatohepatitis.


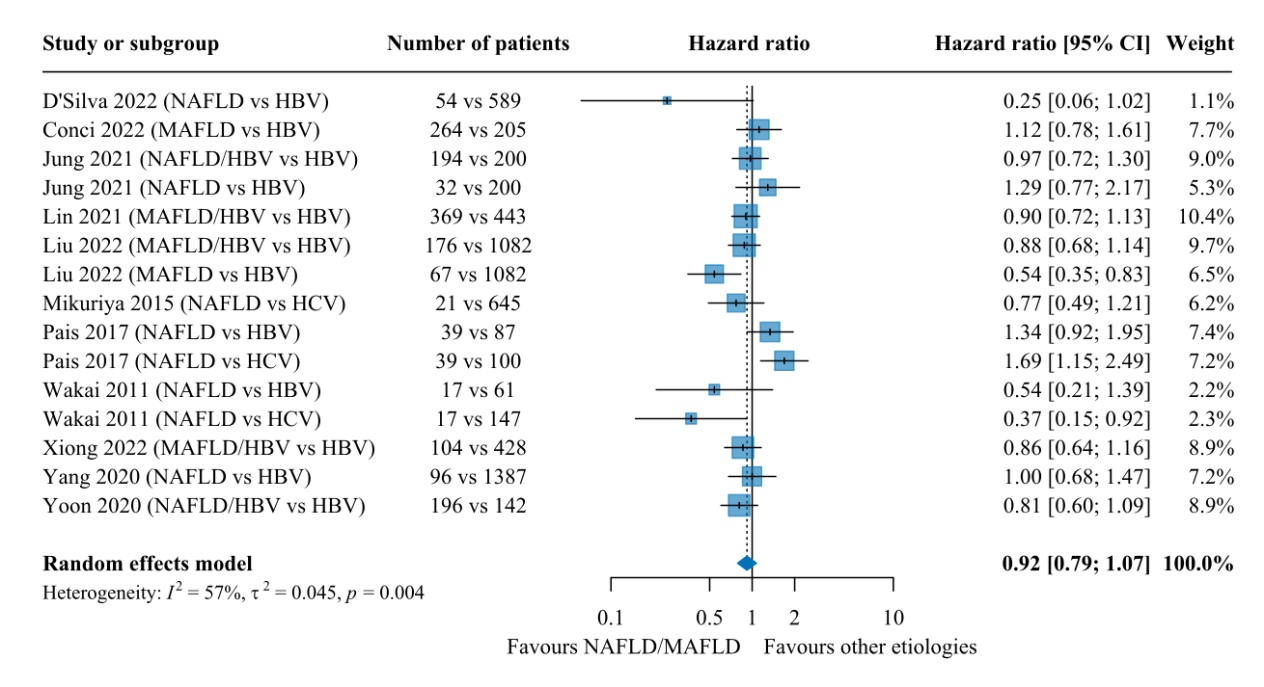

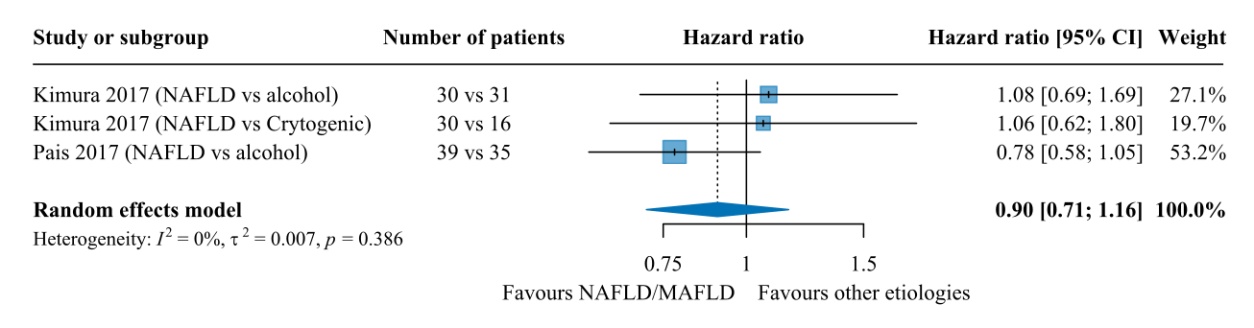


**Figure S6.** Comparison of recurrence-free survival between patients with hepatocellular carcinoma (HCC) related to non-alcoholic fatty liver disease (NAFLD) or metabolic-associated fatty liver disease (MAFLD), and those with HCC of other etiologies among the subsets of (up) HBV or HCV related HCC or (down) those with HCC related to alcoholic or cryptogenic cirrhosis.

HBV, hepatitis B virus; HCV, hepatitis C virus; MAFLD, metabolic-associated fatty liver disease; NAFLD, non-alcoholic fatty liver disease; NASH, nonalcoholic steatohepatitis.


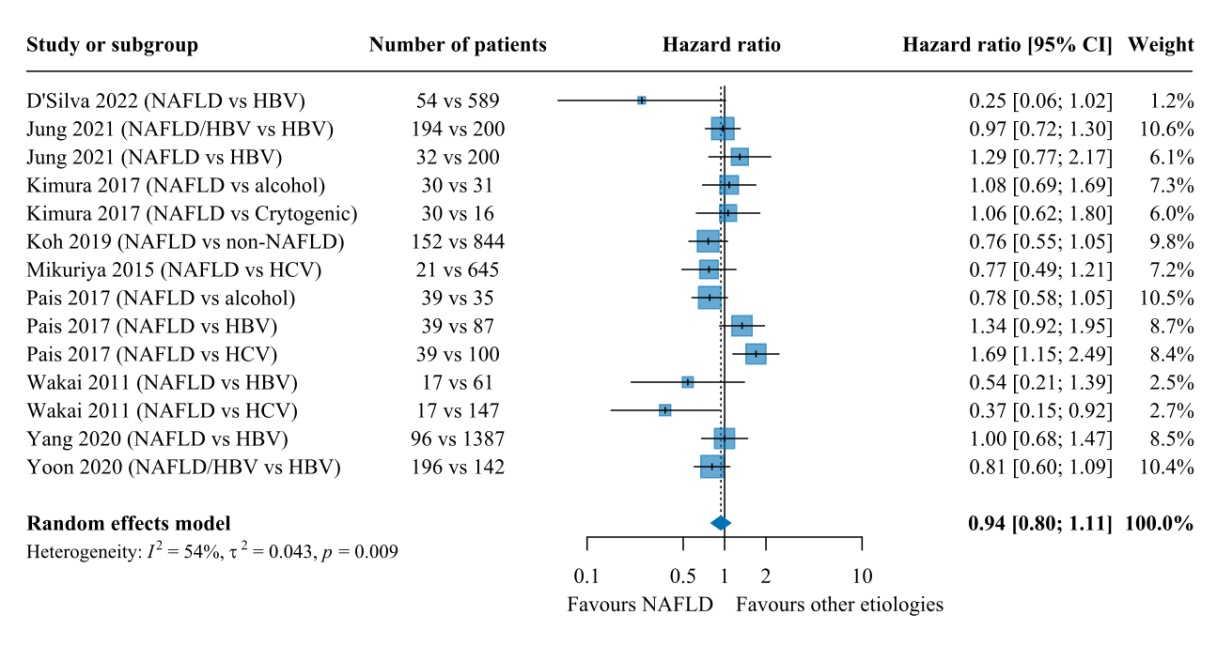


**Figure S7.** Comparison of recurrence-free survival between patients with hepatocellular carcinoma (HCC) related to non-alcoholic fatty liver disease (NAFLD), and those with HCC of other etiologies.

HBV, hepatitis B virus; HCV, hepatitis C virus; NAFLD, non-alcoholic fatty liver disease; NASH, nonalcoholic steatohepatitis.


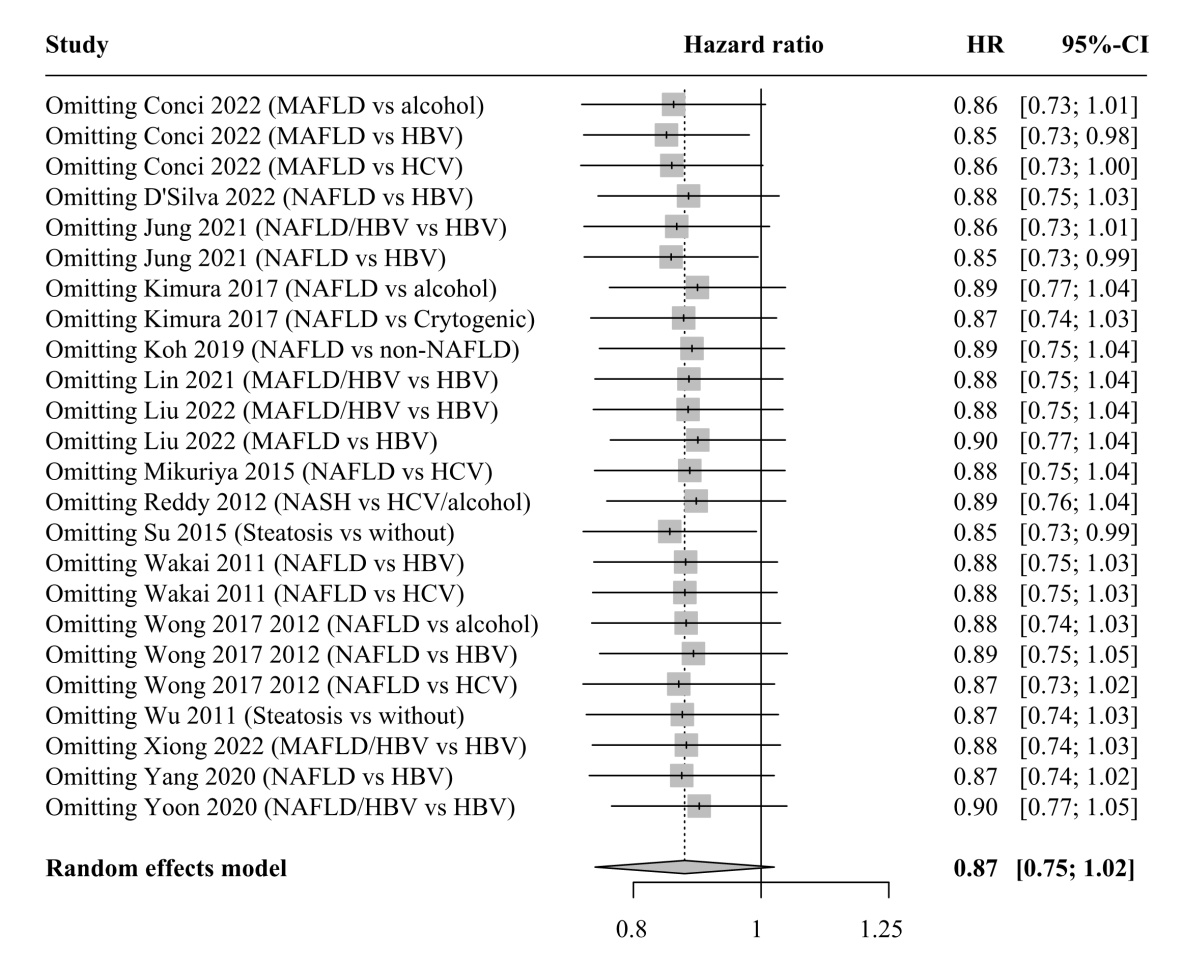

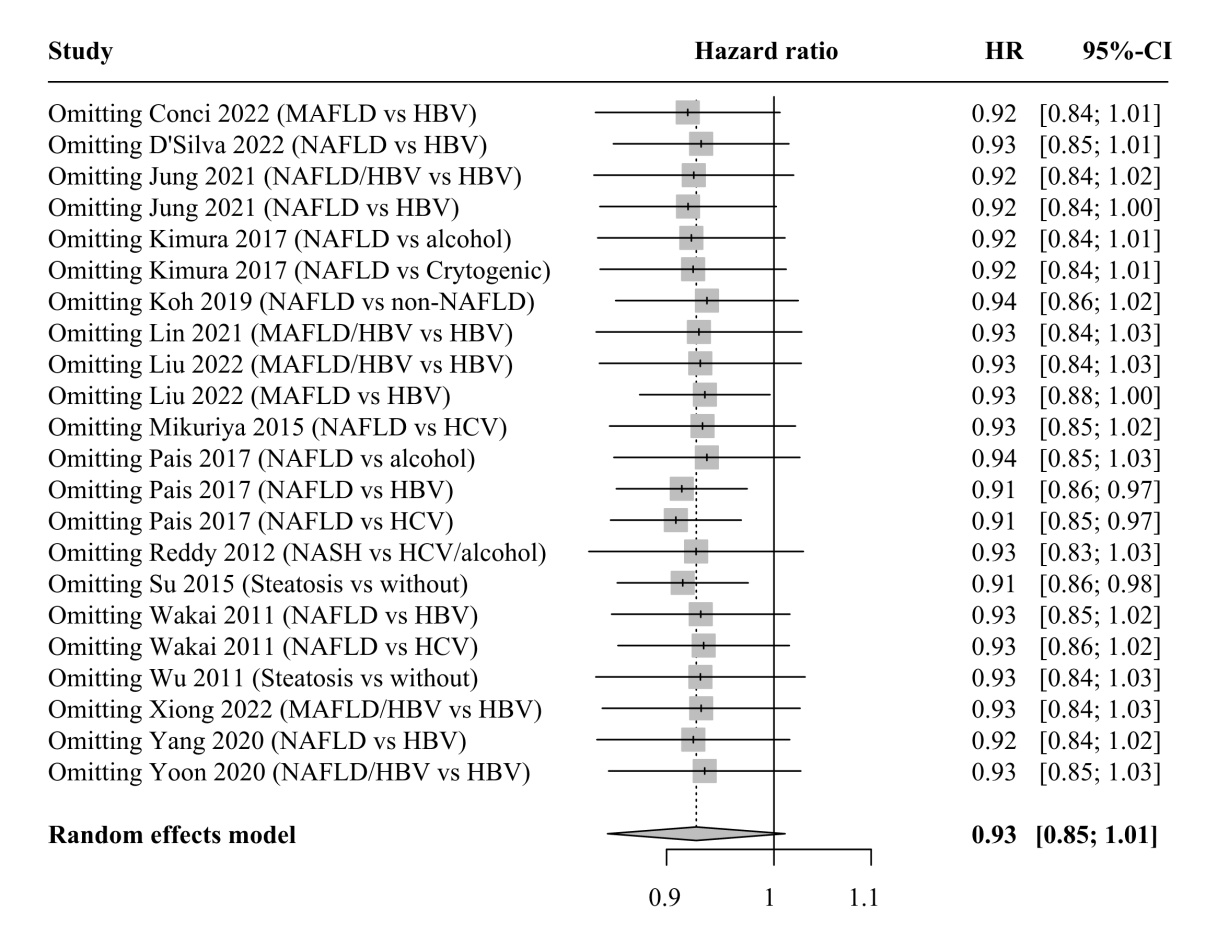


**Figure S8.** Sensitivity analysis (“leave-one-out”) on the meta-analysis of (up) overall survival or (down) recurrence-free survival in the entire cohort.


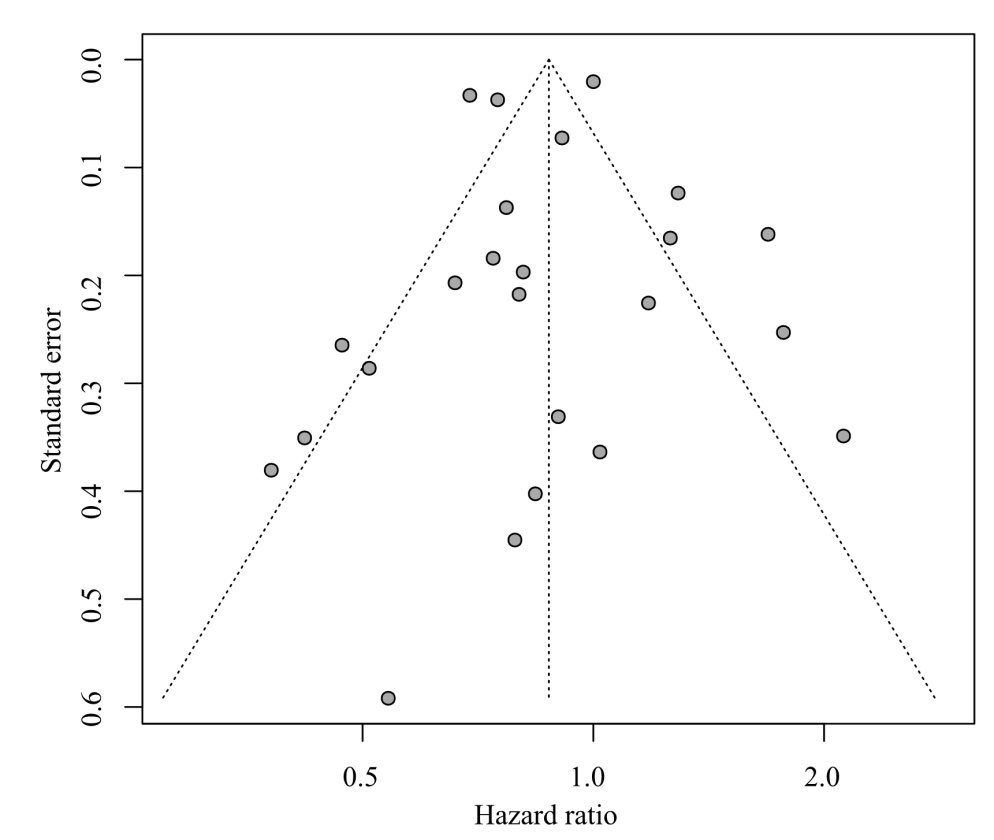

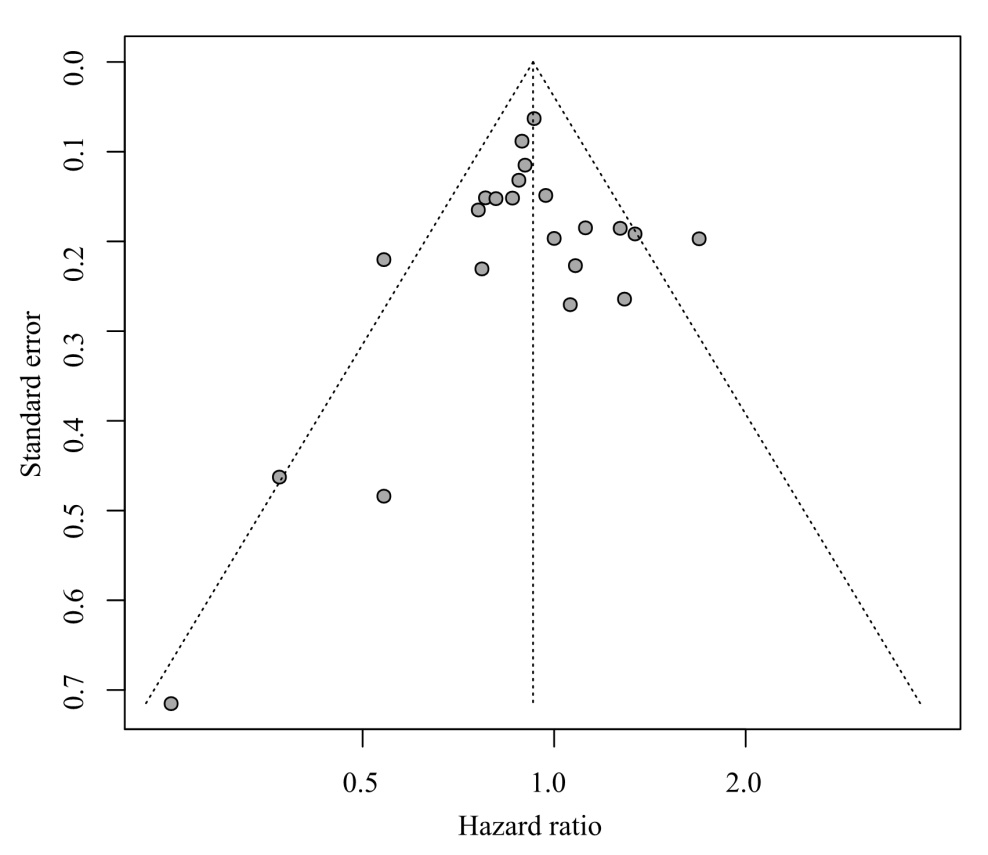


**Figure S9.** Publication bias in the meta-analysis of (up) overall survival or (down) recurrence-free survival in the entire cohort, as estimated by Begg’s test.

**Table S1:** QA assessment result.

|  | Selection | | | | Comparability | Outcome | | | Total score |
| --- | --- | --- | --- | --- | --- | --- | --- | --- | --- |
| Author, year | **Representativeness of the exposed cohort** | **Selection of the non-exposed cohort** | **Ascertainment of exposure** | **Outcome not present at start of study** | **Comparability of cohorts on the basis of the design or analysis** | **Assessment of outcome** | **Follow-up long enough for outcomes to occur** | **Adequacy of follow-up of cohorts** |  |
| Liu 2022^19^ | B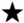 | A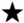 | A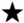 | A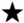 | A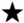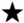 | A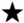 | A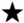 | A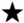 | 9 |
| Conci 2022^20^ | A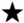 | A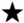 | A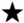 | A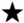 | A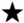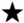 | A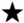 | A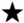 | A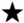 | 9 |
| Jung 2021^21^ | C | A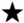 | A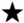 | A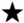 | A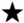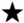 | A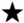 | A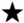 | A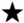 | 8 |
| Lin 2021^22^ | A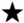 | A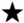 | A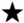 | A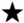 | B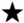 | A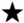 | A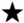 | A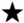 | 8 |
| Kimura 2017^48^ | C | A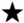 | A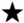 | A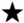 | B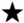 | A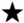 | A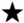 | A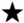 | 7 |
| Koh 2019^49^ | A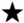 | A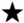 | A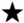 | A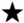 | A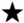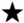 | A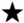 | A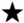 | A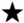 | 9 |
| Mikuriya 2015^50^ | C | A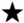 | A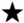 | A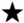 | A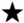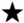 | A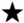 | A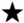 | A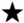 | 8 |
| Pais 2017^51^ | B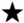 | A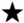 | A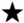 | A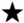 | B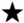 | A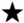 | A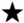 | A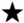 | 8 |
| Reddy 2012^52^ | C | A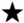 | A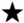 | A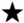 | B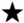 | A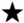 | A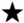 | A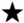 | 7 |
| Su 2015^53^ | C | A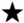 | A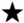 | A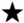 | B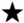 | A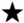 | B | B | 5 |
| Wakai 2011^54^ | C | A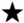 | A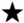 | A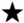 | B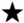 | A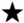 | A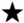 | A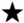 | 7 |
| Wong 2017^55^ | A | A | A | A | B | B | A | A | 8 |
| Wu 2011^56^ | B | A | A | A | B | A | A | A | 8 |
| Yang 2020^57^ | B | A | A | A | A | A | A | A | 9 |
| Yoon 2020^58^ | B | A | A | A | A | A | A | A | 9 |
| D'Silva 2022^59^ | C | A | A | A | B | A | A | A | 7 |
| Xiong 2022^60^ | B | A | A | A | B | A | A | A | 8 |

**Table S2.** Comparison of prognosis of patients with NAFLD-related HCC or HCC of other etiologies (only some cases received hepatectomy).

| **Studies** | **Country/region** | **Inclusion period** | **Etiologies of HCC** | **Sample size** | **Outcomes** |
| --- | --- | --- | --- | --- | --- |
| Lin 2021^29^ | Taiwan | 2011-2019 | NAFLD | 23 | Similar overall survival |
|  |  |  | HBV | 156 |  |
| Piscaglia 2016^30^ | Italy | 2010-2012 | NAFLD | 145 | Similar overall survival |
|  |  |  | HCV | 611 |  |
| Kumar 2020^31^ | Singapore | 2000-2013 | NASH | 54 | Similar overall survival |
|  |  |  | Alcohol | 45 |  |
| Weinmann 2015^32^ | Germany | 2000-2010 | NASH | 45 | Similar overall survival |
|  |  |  | Non-NASH | 1074 |  |
| Tokushige 2010^33^ | Japan | 1990-2007 | NASH | 34 | Similar overall survival |
|  |  |  | HCV | 56 |  |

HBV, hepatitis B virus; HCV, hepatitis C virus; NAFLD, non-alcoholic fatty liver disease; NASH, nonalcoholic steatohepatitis; OS, overall survival.

**References**

[19] Liu L, Xie S, Teng YX, et al. Outcomes of hepatectomy for metabolic dysfunction-associated fatty liver disease or Chronic Hepatitis B-related HCC. *Front Oncol.* 2022;11(1):783339.

[20] Conci S, Cipriani F, Donadon M, et al. Hepatectomy for Metabolic Associated Fatty Liver Disease (MAFLD) related HCC: Propensity case-matched analysis with viral- and alcohol-related HCC. *Eur J Surg Oncol.* 2022;48(1):103-112.

[21] Jung YB, Yoo JE, Han DH, et al. Clinical and survival outcomes after hepatectomy in patients with non-alcoholic fatty liver and hepatitis B-related hepatocellular carcinoma. *HPB (Oxford).* 2021;23(7):1113-1122.

[22] Lin YP, Lin SH, Wang CC, et al. Impact of MAFLD on HBV-Related Stage 0/A Hepatocellular Carcinoma after Curative Resection. *J Pers Med.* 2021;11(8).

[29] Lin BZ, Lin TJ, Lin CL, et al. Differentiation of clinical patterns and survival outcomes of hepatocellular carcinoma on hepatitis B and nonalcoholic fatty liver disease. *J Chin Med Assoc.* 2021;84(6):606-613.

[30] Piscaglia F, Svegliati-Baroni G, Barchetti A, et al. Clinical patterns of hepatocellular carcinoma in nonalcoholic fatty liver disease: A multicenter prospective study. *Hepatology.* 2016;63(3):827-838.

[31] Kumar R, Goh BG, Kam JW, Chang PE, Tan CK. Comparisons between non-alcoholic steatohepatitis and alcohol-related hepatocellular carcinoma. *Clin Mol Hepatol.* 2020;26(2):196-208.

[32] Weinmann A, Alt Y, Koch S, et al. Treatment and survival of non-alcoholic steatohepatitis associated hepatocellular carcinoma. *BMC Cancer.* 2015;15:210.

[33] Tokushige K, Hashimoto E, Yatsuji S, et al. Prospective study of hepatocellular carcinoma in nonalcoholic steatohepatitis in comparison with hepatocellular carcinoma caused by chronic hepatitis C. *J Gastroenterol.* 2010;45(9):960-967.

[48] Kimura T, Kobayashi A, Tanaka N, et al. Clinicopathological characteristics of non-B non-C hepatocellular carcinoma without past hepatitis B virus infection. *Hepatol Res.* 2017;47(5):405-418.

[49] Koh YX, Tan HJ, Liew YX, et al. Liver Resection for Nonalcoholic Fatty Liver Disease-Associated Hepatocellular Carcinoma. *J Am Coll Surg.* 2019;229(5):467-478.e461.

[50] Mikuriya Y, Tashiro H, Kobayashi T, et al. Clinicopathological features of hepatocellular carcinoma in patients with nonalcoholic fatty liver disease. *Langenbecks Arch Surg.* 2015;400(4):471-476.

[51] Pais R, Fartoux L, Goumard C, et al. Temporal trends, clinical patterns and outcomes of NAFLD-related HCC in patients undergoing liver resection over a 20-year period. *Aliment Pharmacol Ther.* 2017;46(9):856-863.

[52] Reddy SK, Steel JL, Chen HW, et al. Outcomes of curative treatment for hepatocellular cancer in nonalcoholic steatohepatitis versus hepatitis C and alcoholic liver disease. *Hepatology.* 2012;55(6):1809-1819.

[53] Su CW, Chau GY, Hung HH, et al. Impact of Steatosis on Prognosis of Patients with Early-Stage Hepatocellular Carcinoma After Hepatic Resection. *Ann Surg Oncol.* 2015;22(7):2253-2261.

[54] Wakai T, Shirai Y, Sakata J, Korita PV, Ajioka Y, Hatakeyama K. Surgical outcomes for hepatocellular carcinoma in nonalcoholic fatty liver disease. *J Gastrointest Surg.* 2011;15(8):1450-1458.

[55] Wong CR, Njei B, Nguyen MH, Nguyen A, Lim JK. Survival after treatment with curative intent for hepatocellular carcinoma among patients with vs without non-alcoholic fatty liver disease. *Aliment Pharmacol Ther.* 2017;46(11-12):1061-1069.

[56] Wu TH, Yu MC, Chan KM, et al. Prognostic effect of steatosis on hepatocellular carcinoma patients after liver resection. *Eur J Surg Oncol.* 2011;37(7):618-622.

[57] Yang T, Hu LY, Li ZL, et al. Liver Resection for Hepatocellular Carcinoma in Non-alcoholic Fatty Liver Disease: a Multicenter Propensity Matching Analysis with HBV-HCC. *J Gastrointest Surg.* 2020;24(2):320-329.

[58] Yoon JS, Lee HY, Chung SW, et al. Prognostic impact of concurrent nonalcoholic fatty liver disease in patients with chronic hepatitis B-related hepatocellular carcinoma. *J Gastroenterol Hepatol.* 2020;35(11):1960-1968.

[59] D'Silva M, Cho JY, Han HS, et al. Long-term surgical outcomes of Non alcoholic fatty liver disease associated hepatocellular carcinoma. *Surg Oncol.* 2022;41:101730.

[60] Xiong KG, Ke KY, Chen LF, et al. The impact of metabolic dysfunction-associated fatty liver disease on the prognosis of patients with hepatocellular carcinoma after radical resection. *Hepatobiliary Pancreat Dis Int.* 2022:doi: 10.1016/j.hbpd.2022.1004.1001. Online ahead of print.
